# Variants in *IRF5* Increase the Risk of Primary Sjögren’s Syndrome in the Mexican Population

**DOI:** 10.3390/ijms27020599

**Published:** 2026-01-07

**Authors:** Julian Ramírez-Bello, Isaac Alberto López-Briceño, Guillermo Valencia-Pacheco, Rosa Elda Barbosa-Cobos, Gabriela Hernández-Molina, Silvia Jiménez-Morales, Iván Sammir Aranda-Uribe, Isela Montúfar-Robles, Swapan K. Nath

**Affiliations:** 1 Subdirección de Investigación Clínica, Instituto Nacional de Cardiología Ignacio Chávez, México City 14080, Mexico; 2 Laboratorio de Hematología, Centro de Investigaciones Regionales “Dr. Hideyo Noguchi”, Universidad Autónoma de Yucatán, Mérida 97069, Mexico; 3 Servicio de Reumatología, Hospital Juárez de México, México City 07760, Mexico; 4 Departamento de Inmunología y Reumatología, Instituto Nacional de Ciencias Médicas y Nutrición Salvador Zurbirán, México City 14080, Mexico; 5 Laboratorio de Innovación y Medicina de Precisión, Núcleo “A”, Instituto Nacional de Medicina Genómica, México City 14610, Mexico; 6 Laboratorio de Biología Celular y Molecular, Departamento de Ciencias Farmacéuticas, División de Ciencias de la Salud, Universidad Autónoma de Quintana Roo, Chetumal 77019, Mexico; 7 División de Investigación, Hospital Juárez de México, México City 07760, Mexico; 8 Arthritis and Clinical Immunology Research Program, Oklahoma Medical Research Foundation, Oklahoma City, OK 73104, USA

**Keywords:** primary Sjögren’s syndrome, *IRF5*, genetic variants, susceptibility

## Abstract

Primary Sjögren’s syndrome (pSS) is an autoimmune disease characterized by inflammation and damage to salivary and lacrimal glands. Its etiology involves both genetic and environmental factors. Among susceptibility genes, *IRF5* has been highlighted in European populations, but evidence in non-European groups remains limited. This study evaluated whether *IRF5* variants rs2004640G/T, rs2070197T/C, rs10954213G/A, and rs59110799G/T are associated with pSS susceptibility, clinical manifestations, or the presence of autoantibodies in a Mexican population. The diagnosis was confirmed by rheumatologists using the 2016 ACR–EULAR classification criteria for pSS. Genotyping was performed using TaqMan probes in 231 controls and 132 pSS patients from central Mexico. Associations were analyzed through binary logistic regression under different genetic models, adjusting for age and geographic origin. Clinical correlations were examined with SNPStats, and haplotypes were constructed using Haploview. Results showed that all four *IRF5* variants were significantly associated with pSS susceptibility. Moreover, rs2004640, rs2070197, and rs10954213 variants were associated with arthritis, a frequent clinical manifestation in pSS patients. This represents the first evidence in a Latin American population demonstrating that *IRF5* variants contribute to increased risk of developing pSS. These findings suggest ethnicity-specific genetic influences and highlight the importance of expanding research beyond European cohorts. Replication in larger samples and functional analyses are needed to confirm these associations and clarify their biological relevance.

## 1. Introduction

Primary Sjögren’s syndrome (pSS) is a complex autoimmune disease (AD) characterized by dysfunction and destruction of exocrine glands due to lymphocytic infiltration and immune hyperreactivity [1]. In addition to glandular involvement, extra-glandular manifestations of pSS include arthritis, Raynaud’s phenomenon, vasculitis, neuropathy, and multi-organ involvement [2]. The prevalence of pSS in the general population is estimated at 0.6%. Like most ADs, women are more commonly affected, with a female-to-male ratio of 9:1 [3,4]. The exact cause of pSS remains unclear; however, studies indicate that it is a multifactorial disease involving genetic, hormonal and environmental factors, which contribute to its onset and progression [5].

Research into the pathogenic mechanisms of pSS has highlighted the central role of interferons (IFNs) in disease development. Specifically, some studies have identified plasmacytoid dendritic cells (pDC)—IFN-producing cells—in the salivary glands of pSS patients [6,7]. Furthermore, pSS shares numerous pathogenic features with systemic lupus erythematosus (SLE), indicating overlapping immune pathways. These shared characteristics include the production of anti-SSA/SSB autoantibodies, activation of interferon (IFN) signaling pathways, recruitment of plasmacytoid dendritic cells (pDCs), and predisposing genetic factors [8]. Additionally, single-nucleotide variants (SNVs) in genes involved in IFN signaling have been strongly associated with SLE, particularly those within the *IRF5* gene, which encodes interferon regulatory factor 5 [9]. In mammals, the IRF family comprises 10 members (IRF1–IRF10), which are essential regulators of immune cell activation and function. Along with pattern recognition receptors, IRFs link innate and adaptive immune responses, coordinating antiviral defenses and inflammatory signaling [10].

IRF5 is a transcription factor that plays a crucial role in the Toll-like receptor (TLR) signaling pathway and activates genes that encode inflammatory cytokines [11]. Variants in the *IRF5* gene have been associated with an increased risk of developing various ADs, including SLE [12,13], multiple sclerosis [14], pSS [15], systemic sclerosis [16], and Crohn’s disease [17]. *IRF5* was identified as a susceptibility gene for pSS through genome-wide association studies (GWASs) [18,19,20]. Previously, it had been recognized as one of the main genes associated with SLE susceptibility across Caucasian, Asian, and African populations [21,22].

In the Hispanic population, the *IRF5* gene has been identified as a primary genetic risk *locus* for SLE [23,24]. Our group has also reported associations between *IRF5* variants and SLE susceptibility in two distinct Mexican populations [13]. In the context of pSS, Miceli-Richard et al. reported a significant association between the rs2004640G/T variant and pSS in French patients of Caucasian ancestry; however, no associations were detected for rs10954213G/A or rs2070197T/C [25]. Additionally, the rs59110799G/T variant has been associated with pSS at the GWAS level in Caucasian populations, but not in Asian cohorts [19]. Given the established association of *IRF5* with SLE in the Hispanic population and the close pathogenic relationship between SLE and pSS, our study aimed to determine whether four *IRF5* SNVs (rs2004640G/T, rs2070197T/C, rs10954213G/A, and rs59110799G/T) are associated with pSS susceptibility in a Mexican population.

## 2. Result and Discussion

### 2.1. Clinical Characteristics of pSS Patients

We collected clinical, serological, and comorbidity data from 102 pSS patients. All patients were diagnosed with pSS not associated with other connective tissue diseases; other autoimmune rheumatic conditions, including rheumatoid arthritis, were ruled out. Anti-Ro antibodies were detected in 88.2% of patients, and anti-La antibodies in 52%. The most common extra-glandular manifestation was arthritis, defined as clinical synovitis, non-erosive on hand X-ray, as assessed by rheumatologists, followed by Raynaud’s phenomenon. Ocular symptoms were more frequent than oral symptoms. Data on type 2 diabetes, hypertension, dyslipidemia, and smoking status are summarized in Table 1.

### 2.2. HWE and Statistical Power

The genotypic distributions of the *IRF5* SNVs in the control group were consistent with HWE, rs2004640G/T (*p* = 0.43), rs2070197T/C (*p* = 0.69), rs10954213G/A (*p* = 0.34), and rs59110799G/T (*p* = 0.39). The statistical power of our study was below 80%. Therefore, the results should be interpreted with caution, particularly for the variants rs2070197T/C, rs10954213G/A, and rs59110799G/T, which showed *p*-values close to 0.05 and may be considered borderline associations.

### 2.3. Genotypic and Allelic Frequencies of IRF5 SNVs in pSS Patients and Controls and Association Analysis

The *p*-values for the *IRF5* SNVs in cases and controls were obtained using binary logistic regression and subsequently corrected using the false discovery rate (FDR) method (Table 2) to account for multiple testing. This approach helps to reduce spurious associations while preserving adequate statistical power. Therefore, FDR-adjusted *p*-values provide more reliable evidence for true genetic associations. Our data suggest that the four *IRF5* SNVs—rs2004640G/T, rs2070197T/C, rs10954213G/A, and rs59110799G/T—are associated with an increased risk of developing pSS under several genetic models. Our analysis showed that four *IRF5* SNVs—rs2004640G/T, rs2070197T/C, rs10954213G/A, and rs59110799G/T—were significantly associated with a higher risk of developing pSS in various genetic models. The rs2004640G/T variant was associated with pSS in the allelic (G vs. T, OR = 2.06, *p* = 0.0008), codominant (GG vs. TT, OR = 4.25, *p* = 0.0007), dominant (GG vs. GT + TT, OR = 2.00, *p* = 0.04), and recessive (GG + GT vs. TT, OR = 3.78, *p* = 0.0002) models. The rs2070197T/C was associated under allelic (T vs. C, OR = 1.71, *p* = 0.01), codominant (TT vs. CC, OR = 3.41, *p* = 0.01), and recessive (TT + TC vs. CC, OR = 2.95, *p* = 0.01) models. The rs10954213G/A was associated in allelic (G vs. A, OR = 1.55, *p* = 0.04) and recessive (GG + GA vs. AA, OR = 2.30, *p* = 0.01) models. Finally, the rs59110799G/T variant showed associations in allelic (G vs. T, OR = 1.66, *p* = 0.02), codominant (GG vs. GT, OR = 2.24, *p* = 0.02), and dominant (GG vs. GT + TT, OR = 2.21, *p* = 0.009) models (Table 2). The most robust genetic association was observed for the rs2004640G/T variant, which represents the primary model of association with susceptibility to pSS in our study. Because the statistical power of our study was below 80%, the results should be interpreted with caution, as limited power may increase the likelihood of chance findings (false positives), inflate effect size estimates, and reduce reproducibility [26]. Therefore, our findings should be validated by independent studies including equal or larger numbers of patients and controls with similar ancestral backgrounds.

### 2.4. Association Analysis Between IRF5 SNV, Clinical Features and Autoantibodies in pSS Patients

Clinical trait analyses showed an association between the *IRF5* variants rs2004640G/T, rs2070197T/C, and rs10954213G/A and susceptibility to developing arthritis in patients with pSS after adjustment for age and place of birth. Under the dominant genetic model, these three *IRF5* SNVs showed the following associations: rs2004640G/T (OR = 5.1, *p* = 0.037), rs2070197T/C (OR = 4.6, *p* = 0.005), and rs10954213G/A (OR = 8.1, *p* = 0.049). In addition, these variants also showed associations under other genetic models. The following results were derived from genotype frequency analyses of the *IRF5* variants in pSS patients stratified by the presence or absence of arthritis. For rs2004640G/T, genotype frequencies among patients with arthritis (*n* = 30) were GG: *n* = 2 (6.7%), GT: *n* = 11 (36.7%), and TT: *n* = 17 (56.6%) compared with patients without arthritis, who showed GG: *n* = 20 (27.8%), GT: *n* = 24 (33.3%), and TT: *n* = 28 (38.9%). For rs2070197T/C, genotype frequencies in pSS patients and arthritis were TT: *n* = 5 (16.7%), TC: *n* = 19 (63.3%), and CC: *n* = 6 (20%), whereas patients without arthritis presented TT: *n* = 36 (50.0%), TC: *n* = 21 (29.2%), and CC: *n* = 15 (20.8%). Regarding rs10954213G/A, pSS patients and arthritis showed genotype frequencies of GG: *n* = 1 (3.3%), GA: *n* = 14 (46.7%), and AA: *n* = 15 (50.0%), while patients without arthritis exhibited GG: *n* = 17 (23.6%), GA: *n* = 25 (34.7%), and AA: *n* = 30 (41.7%). However, after correction of the *p*-values using the FDR method, the associations between these *IRF5* variants and arthritis in patients with pSS were no longer statistically significant. No statistically significant associations were found between any of the *IRF5* variants and the presence of anti-Ro or anti-La antibodies. It is important to note that, due to the small sample size, our findings should be considered exploratory. Although the data suggest an association between these three *IRF5* variants and arthritis after adjustment for age and place of birth, the results should be interpreted with caution, as the association was no longer significant after applying the FDR correction. Therefore, these findings require validation in independent cohorts with similar or different ancestry to better clarify the role of these variants in susceptibility to arthritis among patients with pSS.

### 2.5. LD and Haplotype Analysis

**Figure 1:**
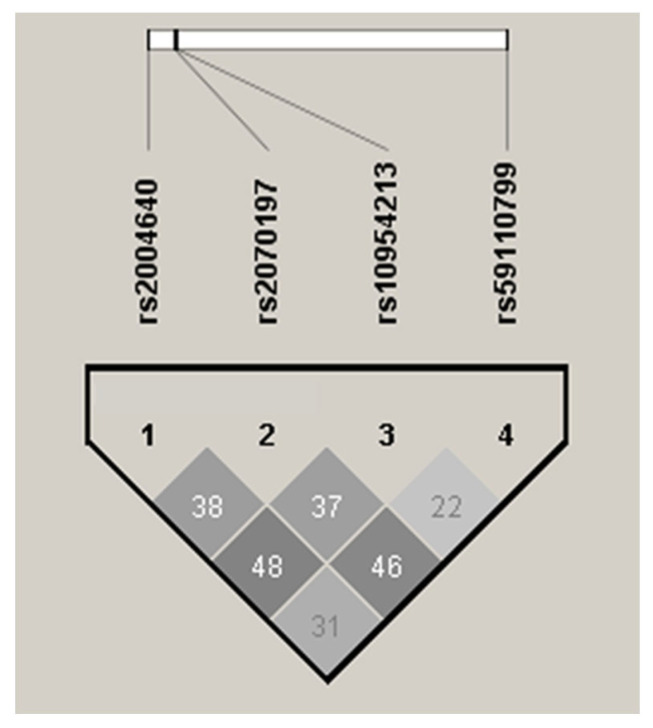
Linkage disequilibrium plot in pSS patients and healthy controls. All pairwise r^2^ values (gray-scale boxes) between *IRF5* SNVs were <0.80, indicating weak linkage disequilibrium.

LD between *IRF5* variants was assessed using the r^2^ metric, and haplotype blocks were defined according to the observed LD structure. Only haplotypes with a frequency greater than 1% in control individuals were included in the association analysis. The statistical significance of haplotype associations was evaluated using permutation testing implemented in Haploview, which allows correction for multiple testing while accounting for the underlying LD among variants. LD analysis of the *IRF5* variants was performed and visualized for both pSS patients and controls (Figure 1). The results showed that none of the variants were in strong LD (r^2^ < 0.8). In our study, seven haplotypes with a frequency greater than 1% were identified in controls. The GTGG haplotype, which carries the common alleles of all four *IRF5* variants, was significantly associated with protection against pSS (Table 3). In contrast, the TTGG haplotype, which includes the T risk allele of rs2004640G/T, was associated with increased susceptibility to this AD (OR = 3.91, *p* = 0.003; Table 3). This association remained statistically significant after permutation analysis using 10,000 permutations. Permutation-based correction accounts for multiple testing, controls type I error, and adjusts for the number of SNVs evaluated in cases and controls. Our haplotype analysis indicates that the T risk allele of the rs2004640G/T variant is the main driver of the association between the TTGG haplotype and susceptibility to pSS. The effect of this allele on disease risk is evident, as the TTGG haplotype remains significantly associated with pSS despite containing three alleles (TGG) from rs2070197T/C, rs10954213G/A, and rs59110799G/T that are individually common and protective. Notably, while the rs2004640T allele alone confers an odds ratio (OR) of 2.06, its inclusion within the TTGG haplotype increases the OR to 3.99, thereby strengthening the association with pSS. In contrast, the TCAT haplotype, which carries the four risk alleles of rs2004640G/T, rs2070197T/C, rs10954213G/A, and rs59110799G/T, did not show a statistically significant association with pSS, although a trend was observed (*p* = 0.06). The OR for this haplotype decreased to 1.58, which is lower than the OR observed for the rs2004640T allele alone. These findings suggest that the rs2004640G/T variant plays a predominant role in pSS susceptibility, either individually or within specific haplotypic contexts. Although the variants rs2070197T/C, rs10954213G/A, and rs59110799G/T showed individual associations with pSS, they did not independently contribute to disease susceptibility when evaluated as part of haplotypes. Overall, while the combination of risk alleles in the TCAT haplotype increases the haplotype OR to 3.91 compared with the individual ORs of each variant (2.06, 1.76, 1.55, and 1.66, respectively), this association did not reach statistical significance, underscoring the dominant contribution of rs2004640T to pSS risk.

Although ADs are a diverse group of chronic disorders, they share several features, including overlapping clinical phenotypes, autoantibodies, dysregulated immune responses, chronic inflammation, a higher prevalence in women, and a strong genetic component [27,28]. One gene commonly and consistently associated with different ADs is *IRF5*, which encodes the interferon regulatory factor 5 [29]. *IRF5* has been identified as a major susceptibility gene for SLE in European and Latin American populations [23,24]; however, its role in pSS appears less significant in individuals of Caucasian ancestry [19]. Notably, a GWAS identified *IRF5* as the primary susceptibility gene for SLE in Hispanic populations [23]. In the Mexican population, several studies have reported associations between *IRF5* variants and SLE susceptibility in both adult and pediatric patients from Mexico City and Yucatán [13,30]. Additionally, published data from our group show an association between *IRF5* SNVs and susceptibility to rheumatoid arthritis (RA) [31]. The present study further expands on these findings by demonstrating that *IRF5* variants are also associated with susceptibility to pSS in this population.

Our results demonstrate an association between the rs2004640T allele and susceptibility to pSS in the Mexican population, which is consistent with the findings of Miceli-Richard, who identified this allele as a risk factor for pSS in French patients [25]. Three distinct IRF5 promoters give rise to several transcripts that include exons 1A, 1B, and 1C. Functional studies have demonstrated that the rs2004640G allele promotes the expression of transcripts containing exons 1A and 1C. In contrast, the rs2004640T allele, which is associated with susceptibility to several ADs, including pSS, drives the expression of all three exons: 1A, 1B, and 1C. This intronic variant, located two base pairs downstream of the intron–exon boundary of exon 1B, introduces a novel 5′ donor splice site within an alternative exon 1. This results in the inclusion of exon 1B in the transcript, generating alternative IRF5 mRNA isoforms. These isoforms are associated with elevated IRF5 expression and increased levels of IFN-α, a key cytokine involved in the pathogenesis of different ADs [32].

Regarding the rs2070197T/C and rs10954213G/A variants, both located in the 3′-untranslated polyadenylation region (3′-UTR) of *IRF5*, our findings contrast with those reported by Miceli-Richard [25], who found no association between these variants and pSS in a French population. In contrast, our data shows an association between both variants and susceptibility to pSS in the Mexican population. These contrasting results underscore the importance of conducting genetic studies across diverse ethnic groups, as genetic susceptibility to ADs can vary significantly by ancestry. To our knowledge, aside from the study by Miceli-Richard et al., no additional studies have specifically addressed the role of these variants in pSS, underscoring the need for further investigations in diverse populations to clarify their potential contribution to disease risk. To our knowledge, no additional studies have evaluated these three *IRF5* variants—rs2004640G/T, rs2070197T/C, and rs10954213G/A—in the context of pSS. Thus, our findings represent the first report to demonstrate an association of rs2070197T/C and rs10954213G/A with pSS susceptibility. It is important to emphasize that these and other *IRF5* variants should be evaluated in populations of diverse ancestries. Additionally, our findings should be replicated in independent cohorts of pSS patients from Mexico or in populations with similar genetic backgrounds. The rs10954213G/A and rs2070197T/C variants have been reported as important genetic factors contributing to SLE susceptibility in various populations, including Caucasians [32,33,34], African Americans [35], and Mexicans [13,30]. However, these associations have not been observed in Asian populations [36], suggesting that ethnic-specific genetic effects may be at play. Our current study contributes novel evidence supporting the role of *IRF5* rs10954213G/A and rs2070197T/C variants in pSS susceptibility, at least within a Latin American population, where *IRF5* has also been identified as the primary SLE susceptibility gene [23]. These findings highlight the relevance of population-specific studies in elucidating the genetic architecture of ADs.

Functional studies demonstrated that the rs10954213A allele generates an IRF5 mRNA isoform with a shortened 3′-untranslated region (3′-UTR) by creating an alternative polyadenylation signal. This allele introduces a canonical hexamer sequence (AAUAAA)—the standard motif for pre-mRNA cleavage and polyadenylation—within the IRF5 3′-UTR. The resulting shorter transcript exhibits increased stability and enhanced expression, leading to elevated levels of *IRF5* mRNA and protein, particularly upon IFNα stimulation [37], a cytokine that serves as a biomarker in pSS patients. Indeed, several studies have demonstrated overexpression of type I interferon–inducible genes (type I IFN signature) in both peripheral blood and salivary glands of pSS patients [2,6].

On the other hand, although previous studies have reported that the rs2070197T/C and rs59110799G/T variants are not functionally relevant, in silico analyses and data from the Genotype-Tissue Expression (GTEx) Portal suggest that both variants may have a regulatory role. These variants could affect microRNA (miRNA) binding or act as expression quantitative trait loci (eQTLs). For example, our in silico analysis shows that the rs2070197C allele creates a potential binding site for miR-24, a miRNA known to regulate various immune-related cells and molecules [13]. This variant might influence the post-transcriptional regulation of IRF5. Additionally, data from the GTEx Portal indicate that the rs2070197T/C functions as an eQTL, modulating IRF5 expression levels in whole blood (*p* = 1.1 × 10^−13^), in pancreas (*p* = 2.6 × 10^−6^), in lung (*p* = 1.7 × 10^−5^), and others. These findings support the idea that these variants could have functional effects and may indeed be relevant in pSS.

Finally, the rs59110799G/T variant—previously reported to be associated with pSS susceptibility in Caucasian patients [19]—was also associated with pSS susceptibility in the Mexican population. Taylor et al. found an association in Caucasians but not in Asian patients [19]. To our knowledge, only Taylor’s study and the present one have evaluated the role of rs59110799G/T in pSS susceptibility. In our cohort, this variant was associated under allelic, codominant, and dominant models. While previous in silico analyses suggest that rs59110799T/G may not be functionally relevant, data from the GTEx Portal indicate that it acts as an eQTL, modulating IRF5 expression in whole blood (*p* = 3.7 × 10^−14^), in liver (*p* = 1.1 × 10^−5^), in lung (*p* = 1.6 × 10^−5^), in pancreas (*p* = 2.8 × 10^−4^), and others [13]. This observation suggests that rs59110799G/T may influence the expression of specific IRF5 isoforms, thereby enhancing pro-inflammatory cytokine production and type I interferon signaling pathways, which are central to autoimmune diseases such as pSS, RA, and SLE. However, its potential functional role as an eQTL remains to be fully characterized. Experimental approaches, such as CRISPR-based editing or luciferase reporter assays, are needed to validate its cis-regulatory mechanisms and isoform-specific influences.

pSS affects the salivary and lacrimal glands [1]. It has been estimated that approximately 50% of patients with this AD develop extra-glandular manifestations either at diagnosis or during a mean follow-up period of 94 months, with arthritis/arthralgia being the most frequently reported manifestation, along with other systemic features such as Raynaud’s phenomenon [38]. Our findings are consistent with previous reports, showing that articular involvement is the most common extra-glandular manifestation in pSS patients [38,39]. Additionally, we observed a prevalence of anti-SSA and anti-SSB autoantibodies similar to that reported by other studies, with anti-SSA antibodies being more frequent than anti-SSB in our pSS cohort [39]. Although we initially observed an association between three *IRF5* variants and susceptibility to arthritis in pSS patients, this association disappeared after correction of the *p*-values using the FDR approach. Nevertheless, replication of these findings in independent patient cohorts with diverse clinical characteristics—including arthritis-positive cases—and comprehensive serological profiling is warranted to better elucidate the role of these *IRF5* variants and their potential contribution to arthritis in pSS.

Mexico is a highly admixed country with marked regional differences in ancestral composition. For instance, Mexico City has been estimated to comprise approximately 50% Amerindian, 45% European, and 5% African ancestry [40], whereas western and southeastern regions show distinct ancestry profiles [41]. Although *IRF5* variants are well-established risk factors for SLE and RA in European populations [23,24,31], their role in susceptibility to pSS remains poorly characterized, particularly in Hispanic populations. A study by Alarcón-Riquelme et al. [23] identified *IRF5* as a major susceptibility gene for SLE in Latin American and Native American–enriched populations. We subsequently replicated this finding in patients with SLE from central Mexico [13], a region with a comparable proportion of Amerindian and European ancestry [40]. Similar associations were also observed in SLE patients from Yucatán, a southeastern Mexican state characterized by a higher proportion of Amerindian ancestry relative to European ancestry [41]. Together, the study by Alarcón-Riquelme et al. and our own investigations in SLE patients and controls suggest that, although several susceptibility genes show a strong European contribution, Amerindian ancestry also plays an important role in the genetic susceptibility to ADs such as SLE. Although our current study did not include ancestry-informative markers (AIMs), which represents a limitation, our findings nevertheless suggest that *IRF5* is an important susceptibility gene in mestizo pSS patients from central Mexico, a population characterized by a relatively balanced proportion of European and Amerindian ancestry.

In our study, the *IRF5* GTGG haplotype, which carries the common alleles rs2004640, rs2070197, rs10954213, and rs59110799, was significantly associated with protection against pSS. Given that alternative *IRF5* haplotypes have been linked to increased gene expression and a more intense type I interferon signature in several autoimmune diseases, it is plausible that this combination of common alleles represents a functional “no-risk” configuration, consistent with adequate immune activation yet less prone to chronic inflammation.

To contextualize our findings, we compared the frequency of the GTGG haplotype in our population with those reported in Asian and European studies, noting that not all studies include the same set of SNVs. Despite this, a consistent pattern emerges: haplotypes carrying the common alleles of rs2004640 and rs10954213 are associated with protection against autoimmune diseases. In Chinese populations, the GA and GG haplotypes (rs2004640/rs10954213) are more frequent in controls and have been described as protective [42]; in Japan, the GGA haplotype has also been classified as protective [43]. Similarly, in Korea [44] and in European populations (e.g., the GGT haplotype in the Netherlands) [45], haplotypes that include the common allele of rs2004640 show protective effects. In the United States, the GG haplotype (rs2004640/rs10954213) has also been reported as protective [46]. In this context, the GTGG haplotype observed in our Mexican population—which carries the common alleles of rs2004640 and rs10954213—aligns with this international pattern, reinforcing the biological plausibility of its protective effect against pSS.

In our results, although some SNVs showed modest associations, the GTGG haplotype exhibited a more robust protective effect (OR = 0.41, *p* < 1 × 10^−5^). This suggests that the combination of common alleles reflects a complete functional state that could be more stable and reproducible as a genetic marker. Therefore, the GTGG haplotype may have greater potential for future applications in genetic stratification, risk assessment, or predictive modeling in pSS than the isolated analysis of each variant.

We also acknowledge several limitations of our study: (a) our results need validation in another pSS patient cohort from our population or with similar ancestries; (b) its relatively small sample size limits statistical power and warrants expansion in future work; (c) it is also limited by the absence of ancestry-informative markers, potentially introducing confounding due to population stratification; and (d) regarding age, we acknowledge the difference between patients and controls, which is a common limitation in case–control studies of pSS due to its typically late onset [47]. Therefore, age was included as a covariate in all logistic regression analyses to estimate independent genetic effects while controlling for this potential confounder. Nevertheless, residual confounding cannot be completely excluded, and this limitation is now explicitly acknowledged. Accordingly, our findings should be interpreted with caution and validated in independent cohorts with closer age matching. Future research avenues include multi-ancestry meta-analyses and advanced epigenomic studies to further explore the conserved yet variable role of *IRF5* in autoimmune pathogenesis.

## 3. Methods and Materials

### 3.1. Study Population

The present study included 132 female patients diagnosed with pSS and 231 healthy female controls, all aged 18 years or older. The diagnosis was confirmed by rheumatologists at Hospital Juárez de México (HJM) and the Instituto Nacional de Ciencias Médicas y Nutrición Salvador Zubirán (INCMNSZ) using the 2016 ACR–EULAR classification criteria for pSS. These criteria take into account the Schirmer’s I test, the unstimulated whole saliva flow rate, the labial salivary gland biopsy, the ocular staining score, and the presence of anti-Ro/SSA antibodies. The mean age of pSS patients was 64.83 ± 12.58 years, with an average disease duration of 8 years. Patients with a diagnosis of any additional AD were excluded from the study. The control group had a mean age of 50.89 ± 4.44 years and no personal history of chronic autoimmune or inflammatory diseases, including obesity, asthma, or inflammatory bowel disease, among others. Recognizing that the age difference between cases and controls could introduce bias even after statistical adjustment, we performed a sensitivity analysis. All participants were of homogeneous Mexican ethnicity, with at least three generations of Mexican ancestry, and were recruited from central Mexico. As shown in Table 1, the review of medical records allowed the characterization of the clinical features and treatment regimens of patients diagnosed with pSS. This study was conducted in accordance with the ethical principles outlined in the Declaration of Helsinki, and it was approved by the Ethics, Research, and Biosafety Committees of both HJM (Registration No. HJM 020/21-I; approval date: 6 February 2024) and the INCMNSZ (Registration No. IRE-4641; approval date: 7 August 2023). The consent form was distributed to all participants who signed.

### 3.2. Isolation of DNA from the Buffy Coat

Genomic DNA was isolated from peripheral blood leukocytes obtained from the buffy coat after centrifugation at 2500 rpm for 15 min, using a slightly modified version of the standard protocol described by Lahiri et al. [48].

The quality and concentration of the extracted DNA were evaluated with a NanoDrop One/OneC spectrophotometer (Thermo Scientific, Waltham, MA, USA). The DNA was then diluted in nuclease-free water to a final concentration of 10 ng/μL for downstream genotyping analysis.

### 3.3. Genotyping

Genotyping was performed using an allelic discrimination assay with TaqMan^®^ probes (Applied Biosystems, Foster City, CA, USA) and a CFX Opus 96 Real-Time PCR System thermal cycler (Bio-Rad, Hercules, CA, USA), following the manufacturer’s instructions. Four *IRF5* SNVs were analyzed: rs2004640G/T, rs2070197T/C, rs10954213G/A, and rs59110799G/T. The real-time PCR (RT-PCR) cycling conditions were as follows: initial denaturation at 50 °C for 2 min, followed by 95 °C for 8 min, then 40 cycles of denaturation at 95 °C for 15 s and extension at 60 °C for 1 min. To ensure data reliability, 75% of the samples were genotyped in duplicate, yielding a 100% concordance rate, thereby confirming the reproducibility of the genotyping results.

### 3.4. Statistical Analysis

The Hardy–Weinberg equilibrium (HWE) for each *IRF5* SNV was assessed in the control group using SNPStats (https://www.snpstats.net/start.htm, accessed on 2 January 2026). The statistical power of our study was evaluated using the Quanto program (https://keck.usc.edu/biostatistics/software/, accessed on 2 January 2026; Quanto v1.2). The parameters used for power calculation included (a) an unmatched case–control disease model; (b) a single-gene study design; (c) the minor allele frequencies of the four *IRF5* variants; (d) the assumed genetic inheritance model; (e) the population risk for pSS; (f) an odds ratio of 1.5; (g) a statistical power of 80%; and (h) a two-sided type I error rate of 0.05. Comparisons of allelic and genotypic frequencies between cases and controls were conducted using the chi-square test. Haplotype construction was performed using the Haploview program (version 4.0). Binary logistic regression (statsmodels, Python 3.13.6) was used to evaluate the association of rs2004640G/T, rs2070197T/C, rs10954213G/A, and rs59110799G/T under codominant, allelic, dominant, and recessive genetics models, adjusting for age and geographic origin. Odds ratios (ORs), 95% confidence intervals (95% CIs), and *p*-values were calculated. We applied the FDR correction to account for multiple testing and to control the expected proportion of false-positive results, thereby increasing the reliability of the observed associations.

## 4. Conclusions

Our data suggest that the four *IRF5* variants evaluated in this study may act as genetic risk factors for pSS. However, these results should be interpreted with caution, particularly for the rs2070197T/C, rs10954213G/A, and rs59110799G/T variants, which showed borderline associations. In addition, although the association of the rs2004640G/T, rs2070197T/C, and rs10954213G/A variants with susceptibility to arthritis in pSS patients was no longer significant after FDR correction, it would be important for independent research groups to evaluate these findings in order to confirm or refute our observations, especially considering the limited sample size and statistical power of our study.

## Figures and Tables

**Table 1:** Clinical symptoms, autoantibodies, and comorbidities in pSS patients (*n* = 102).

Variable	Frequency	(%)
**Presence of antibodies**		
Anti-SSA (Ro)	90	88.2
Anti-SSB (La)	53	52.0
**Extra-glandular manifestations**		
Fever	9	8.8
Weight loss	4	3.9
Arthritis	30	29.4
Myositis	1	1.0
Vasculitis	6	5.9
Raynaud phenomenon	9	8.8
Interstitial lung disease	5	4.9
**Symptomatic treatment**		
Ocular	93	91.2
Oral	44	43.1
**Comorbidity**		
Diabetes type 2	9	8.8
Hypertension	27	26.5
Dyslipidemia	30	29.4
Smoking	24	23.5

**Table 2:** Genotypic and allelic frequencies of *IRF5* SNVs and association analysis in pSS patients (*n* = 132) and healthy controls (*n* = 231).

Gene SNV	Model	Genotypes or Alleles	pSS *n* (%)	Controls *n* (%)	OR (95% CI)	*p*-Value Adjusted
*IRF5* rs2004640 G/T	Codominant	GG	26 (19.7)	88 (38.1)	-	-
		GT	46 (34.9)	108 (46.8)	1.23 (0.61–2.51)	0.560
		TT	60 (45.4)	35 (15.1)	4.25 (1.95–9.26)	**7 × 10^−4^**
	Allele	G	98 (37.1)	284 (61.5)	-	-
		T	166 (62.9)	178 (38.5)	2.06 (1.38–3.07)	**8 × 10^−4^**
	Dominant	GG	26 (19.7)	88 (38.1)	-	-
		GT + TT	106 (80.3)	143 (61.9)	2.00 (1.05–3.80)	**0.036**
	Recessive	GG + GT	72 (54.6)	196 (84.9)	-	-
		TT	60 (45.4)	35 (15.1)	3.78 (1.95–7.32)	**2 × 10^−4^**
*IRF5* rs2070197 T/C	Codominant	TT	54 (40.9)	128 (55.4)	-	-
		TC	51 (38.6)	87 (37.7)	1.40 (0.75–2.63)	0.290
		CC	27 (20.5)	16 (6.9)	3.41 (1.38–8.44)	**0.010**
	Allele	T	159 (60.2)	343 (74.2)	-	-
		C	105 (39.8)	119 (25.8)	1.71 (1.13–2.60)	**0.012**
	Dominant	TT	54 (40.9)	128 (55.4)	-	-
		CT + CC	78 (59.1)	103 (44.6)	1.75 (0.98–3.12)	0.056
	Recessive	TT + TC	105 (79.6)	215 (93.1)	-	-
		CC	27 (20.4)	16 (6.9)	2.95 (1.25–6.96)	**0.014**
*IRF5* rs10954213 G/A	Codominant	GG	22 (16.7)	58 (25.1)	-	-
		GA	53 (40.1)	123 (53.2)	0.94 (0.44–1.99)	0.860
		AA	57 (43.2)	50 (21.7)	2.20 (0.99–4.90)	0.110
	Allele	G	97 (36.7)	239 (51.7)	-	-
		A	167 (63.3)	223 (48.3)	1.55 (1.03–2.33)	**0.037**
	Dominant	GG	22 (16.7)	58 (25.1)	-	-
		GA + AA	110 (83.3)	173 (74.9)	1.31 (0.65–2.64)	0.440
	Recessive	GG + GA	75 (56.8)	181 (78.3)	-	-
		AA	57 (43.2)	50 (21.7)	2.30 (1.23–4.29)	**0.010**
*IRF5* rs59110799 G/T	Codominant	GG	62 (47.0)	140 (60.6)	-	-
		GT	51 (38.6)	74 (32.0)	2.24 (1.19–4.22)	**0.016**
		TT	19 (14.4)	17 (7.4)	2.11 (0.82–5.46)	0.120
	Allele	G	175 (66.3)	354 (76.6)	-	-
		T	89 (33.7)	108 (23.4)	1.66 (1.09–2.53)	**0.019**
	Dominant	GG	62 (47.0)	140 (60.6)	-	-
		GT + TT	70 (53.0)	91 (39.4)	2.21 (1.23–3.97)	**0.009**
	Recessive	GG + GT	113 (85.6)	214 (92.6)	-	-
		TT	19 (14.4)	17 (7.4)	1.54 (0.62–3.79)	0.350

SNV: single nucleotide variant; pSS: primary Sjögren’s syndrome; OR: odds ratio; CI: confidence interval. The *p*-values shown are those obtained after FDR correction. *p*-values highlighted in bold indicate *IRF5* variants that are associated with susceptibility to developing pSS under different genetic models.

**Table 3:** Haplotype frequency of *IRF5* variants and association analysis in pSS patients (*n* = 132) and controls (*n* = 231) from central Mexico.

Haplotype	pSS 2n (%)	Controls 2n (%)	OR (95% CI)	*pc*
GTGG	73 (27.7)	224 (48.5)	0.41 (0.29–0.56)	<1 × 10^−5^
TTGG	17 (6.4)	8 (1.7)	3.91 (1.66–9.18)	0.003
TCAT	70 (26.5)	86 (18.6)	1.58 (1.10–2.26)	0.060
TTAG	39 (14.8)	43 (9.3)	1.69 (1.06–2.68)	0.119
GTAG	15 (5.7)	49 (10.6)	0.51 (0.28–0.92)	0.123
TCAG	26 (9.9)	28 (6.1)	1.69 (0.97–2.96)	0.339
TTAT	8 (3.0)	11 (2.4)	1.28 (0.51–3.27)	1.00

The haplotypes are composed, from left to right, of the variants rs2004640G/T, rs2070197T/C, rs10954213G/A, and rs59110799G/T. *pc*: corrected *p* value after 100,000 permutations *. pSS: primary Sjögren’s syndrome; OR: odds ratio; CI: confidence interval. * The permutation-based *p*-value correction accounts for multiple testing, controlling for type I error and adjusting for the number of *IRF5* SNVs evaluated in cases and controls.

## Data Availability

The original contributions presented in this study are included in the article. Further inquiries can be directed to the corresponding authors.
